# Antiphospholipid Antibodies and Autoimmune Haemolytic Anaemia: A Systematic Review and Meta-Analysis

**DOI:** 10.3390/ijms21114120

**Published:** 2020-06-09

**Authors:** Paul R.J. Ames, Mira Merashli, Tommaso Bucci, Daniele Pastori, Pasquale Pignatelli, Alessia Arcaro, Fabrizio Gentile

**Affiliations:** 1 Immune Response & Vascular Disease Unit, Nova University, 1150-082 Lisbon, Portugal; 2 Dumfries & Galloway Royal Infirmary, Dumfries, Scotland DG2 8RX, UK; 3 Department of Internal Medicine, Division of Rheumatology, American University of Beirut, Beirut 11-0236, Lebanon; mm116@aub.edu.lb; 4 Division of Allergy and Clinical Immunology, Department of Medicine, University of Salerno, 84084 Salerno, Italy; tombuc@hotmail.it; 5 Prima Clinica Medica, Atherothrombosis Centre, Department of Clinical, Internal Medicine, Anaesthesiologic, & Cardiovascular Sciences, Sapienza University of Rome, 00185 Rome, Italy; daniele.pastori@uniroma1.it (D.P.); pasquale.pignatelli@uniroma1.it (P.P.); 6 Department of Medicine & Health Sciences ‘V.Tiberio’, Universita’ Del Molise, 86100 Campobasso, Italy; alessia.arcaro@unimol.it (A.A.); gentilefabrizio@unimol.it (F.G.)

**Keywords:** antiphospholipid antibodies, autoimmune haemolytic anaemia, antiglobulin test

## Abstract

The relationship between antiphospholipid antibodies (aPL) and autoimmune haemolytic anaemia (AIHA) has never been systematically addressed. The aim of this study is to assess the link between aPL and AIHA in adult systemic lupus erythematosus (SLE) and antiphospholipid syndrome (APS). This study performed an EMBASE/PubMed search from inception to June 2019 and meta-analysis using Peto’s odds ratios. The pooled prevalence (PP) of IgG/IgM anticardiolipin (aCL) and lupus anticoagulant (LA) was greater in AIHA +ve than AIHA −ve patients (34.7% vs. 27.6%, *p* = 0.03; 33.3% vs. 21.8%, *p* < 0.0001; 20.9% vs. 8.3%, *p* = 0.01). The PP of AIHA was greater in: (1) IgG and IgM aCL +ve than −ve patients (21.8% vs. 11.1%, *p* = 0.001 and 18.7% vs. 6.3%, *p* < 0.0001), (2) in SLE related APS than in primary APS patients (22.8% vs. 3.9% *p* < 0.0001), (3) in APS +ve than APS −ve SLE patients (23.2% vs. 8.4%, *p* = 0.01), and (4) in thrombotic APS than non-thrombotic APS/SLE patients (26.8% vs. 10%, *p* = 0.03). The PP of IgG/IgM aCL and LA was greater in DAT +ve than DAT −ve patients (42.4% vs. 12.8%, *p* < 0.0001; 26.2% vs. 12.8%, *p* = 0.03 and 29.2% vs. 15.7%, *p* = 0.004 respectively). It was found that AIHA prevalence is maximal in SLE with aPL/APS, low-moderate in SLE without aPL and minimal in PAPS. Moreover, AIHA is rightly included among the classification criteria for SLE but not for APS/aPL. The significance of an isolated DAT positivity remains unclear in this setting

## 1. Introduction

Recurrent arterial or venous thrombosis and/or obstetric morbidity in the presence and persistence of antiphospholipid antibodies (aPL) detected via immunoassays or clotting tests characterise the antiphospholipid syndrome (APS): APS may be isolated (primary APS, PAPS) or associated with other autoimmune disease (secondary APS), the most common being represented by systemic lupus erythematosus (SLE, lupus-related APS) [1]. Autoimmune haemolytic anaemia (AIHA) develops when antibodies of the host immune system attack autologous red blood cells, usually demonstrated by a positive direct antiglobulin test (DAT), decreasing the patient’s life span and leading to haemolysis. Further, depending on the temperature at which the antibody-red cell reaction occurs, the antibodies can be defined as warm or cold (reviewed in [2]). AIHA with a positive DAT is part of the haematological domain of the EULAR/ACR classification criteria for SLE [3] whereas either an AIHA or a positive DAT in the absence of anemia are part of the clinical and laboratory criteria for SLE [4]. Conversely, AIHA is not part of the criteria for sero-positive or sero-negative APS [5]. We therefore performed a systematic review and meta-analysis to evaluate the extent of the relationship between aPL and AIHA, and the results are presented herein.

## 2. Results

### 2.1. Number of Included Studies

**Figure 1:**
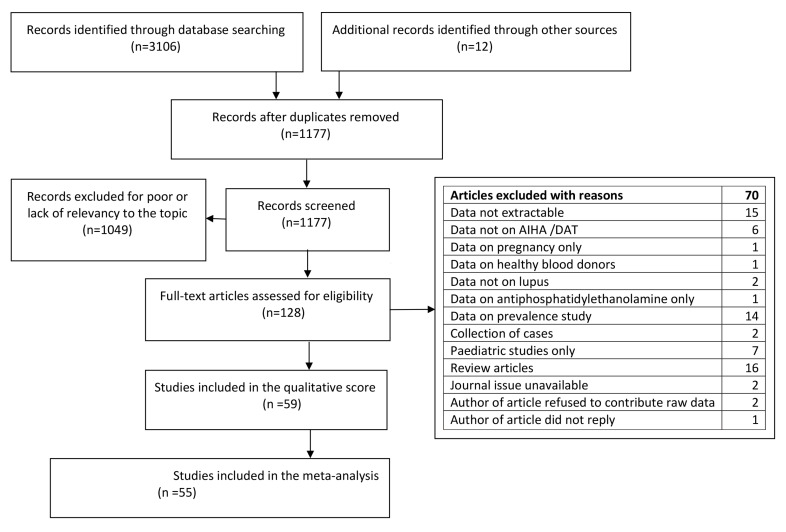
Flow diagram of the literature search according to Prism guidelines.

After completion of the screening and exclusion process (Figure 1), we identified 59 articles [6,7,8,9,10,11,12,13,14,15,16,17,18,19,20,21,22,23,24,25,26,27,28,29,30,31,32,33,34,35,36,37,38,39,40,41,42,43,44,45,46,47,48,49,50,51,52,53,54,55,56,57,58,59,60,61,62,63,64] exploring the relationship between aPL and AIHA/DAT (Table 1) that were included in the qualitative survey. Of these, four were not included in the meta-analysis because three did not share common outcomes with other studies [14,17,29] (one presented mean and standard deviations of the aPL titres) [29] and one presented differential data regarding AIHA in arterial and venous thrombosis that we were not able to discriminate [42].

### 2.2. Clinical Definitions

Of 15 articles having AIHA/DAT as their main topic, 10 provided a clear definition of AIHA [9,19,29,46,47,49,53,57,61] whereas five provided an incomplete or no definition of AIHA [43,48,62,64]. Further, of 42 articles not having AIHA as their main topic, only nine provided a clear definition.

Within the text, we make reference to the pooled prevalence of AIHA, DAT, APL, and thrombosis. This should be intended as pooled prevalence of patients with the given clinical or laboratory feature, but we left the term pooled prevalence to avoid the continuous repetition of the same sentence.

### 2.3. Comparative Prevalence of Antiphospholipid Antibodies in Patients with and without Autoimmune Haemolytic Anaemia

**Figure 2:**
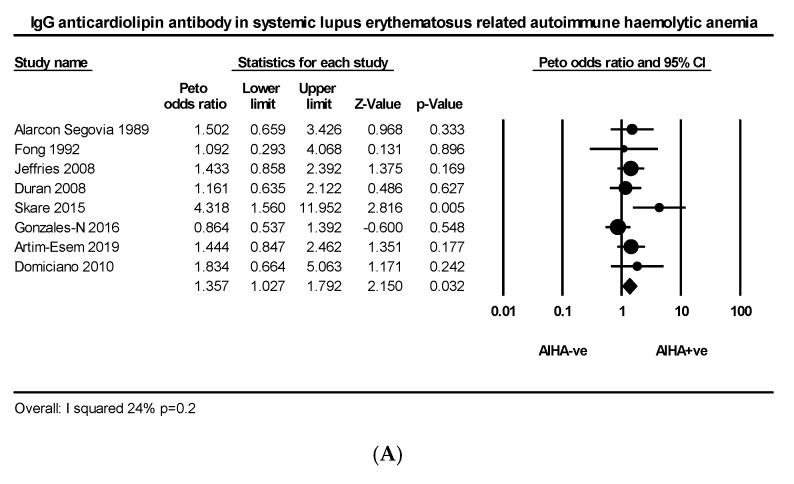

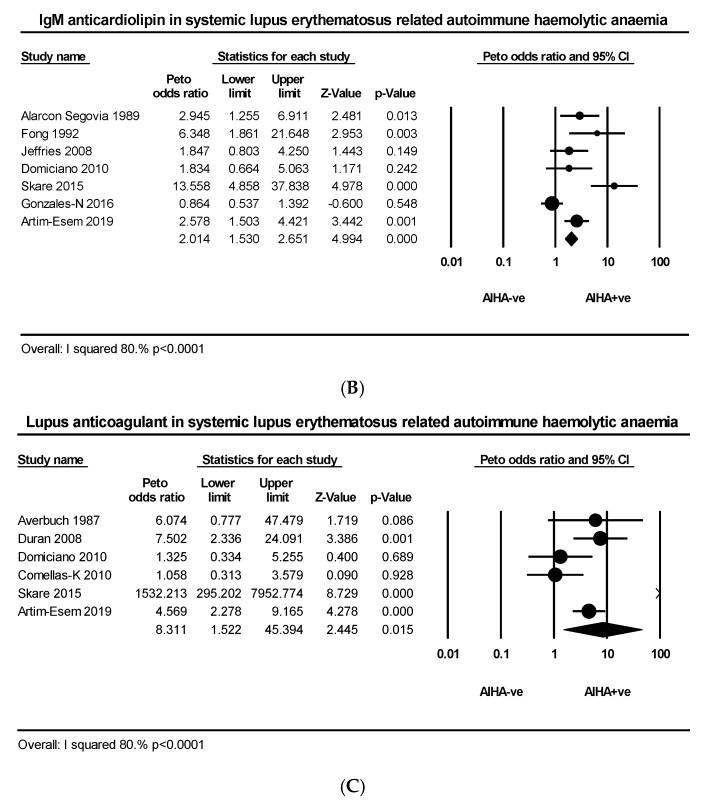
(**A**) Effect size of IgG anticardiolipin, (**B**) IgM anticardiolipin and (**C**) lupus anticoagulant in lupus related autoimmune haemolytic anaemia.

Nine articles comprising 456 AIHA +ve and 3951 AIHA −ve patients explored the relationship between IgG anticardiolipin (aCL) and AIHA in SLE. The PP of IgG aCL was slightly higher in AIHA +ve than AIHA −ve groups (34.6% vs. 28.3%) with mild heterogeneity (*I*^2^ = 24%, *p* = 0.2) (Figure 2A). Eight articles comprising 391 AIHA +ve and 3387 AIHA −ve patients explored the relationship between IgM aCL and AIHA in SLE. The PP of IgM aCL was slightly higher in AIHA +ve than AIHA −ve groups (36% vs. 22%) with wide heterogeneity (*I*^2^ = 80%, *p* < 0.0001) (Figure 2B). Six articles comprising 262 AIHA +ve and 2129 AIHA −ve patients explored the relationship between LA and AIHA in SLE. The PP of LA was greater in AIHA ve than AIHA −ve groups (20.9% vs. 8.3%) with wide heterogeneity (*I*^2^ = 91%, *p* < 0.001 (Figure 2C). Three articles explored the relationship between IgG aCL and idiopathic AIHA, comprising 58 AIHA +ve and 66 AIHA −ve patients; the pooled prevalence of patients with IgG aCL was greater in the AIHA +ve than AIHA −ve group (22.4% vs. 3.3%) (Figure A2).

### 2.4. Comparative Prevalence of Antiphospholipid Antibody in Patients with and without a Positive Coombs Test

**Figure 3:**
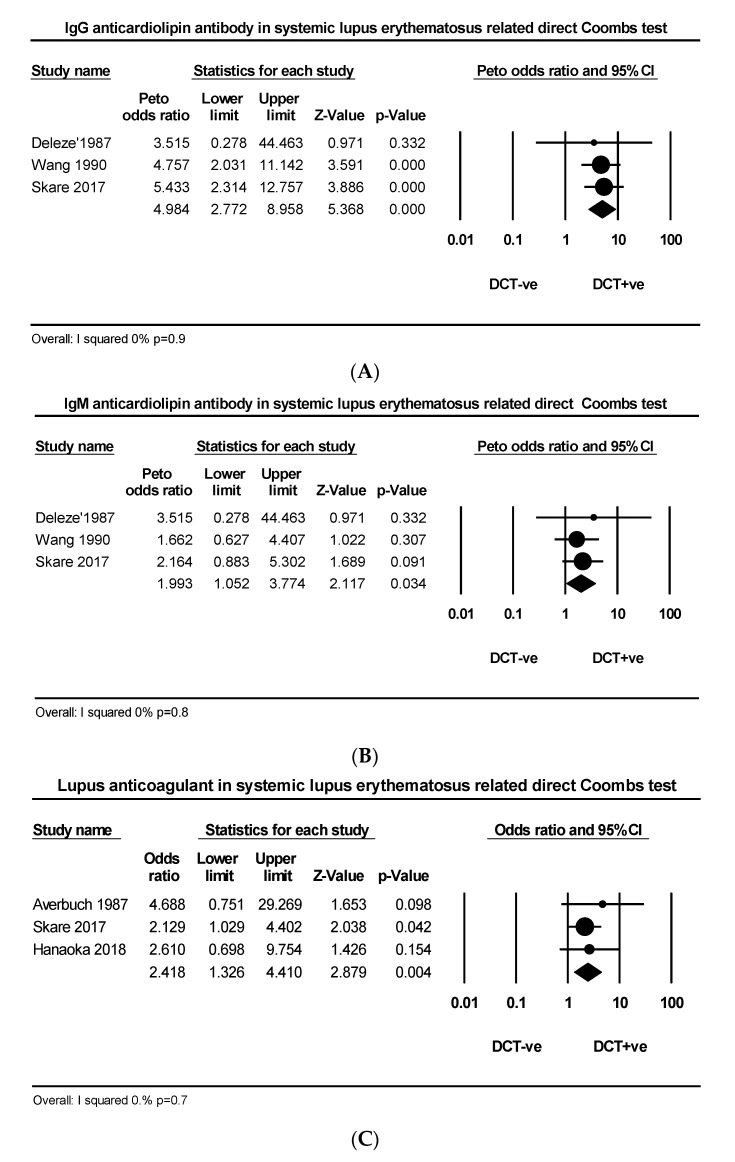
(**A**) Effect size of IgG anticardiolipin, (**B**) IgM anticardiolipin and (**C**) lupus anticoagulant in lupus related direct antiglobulin test.

Three articles comprising 99 DAT +ve and 397 DAT −ve patients explored the relationship between IgG aCL and DAT in SLE. The PP of IgG aCL was much greater in DAT +ve than DAT −ve groups (42.4% vs. 12.8%) (Figure 3A). Three articles comprising 99 DAT +ve and 397 DAT −ve patients explored the relationship between IgM aCL and DAT in SLE. The PP of IgM aCL was greater in DAT +ve than DAT −ve groups (26.2% vs. 12.8%) (Figure 3B). Three articles comprising 65 DAT +ve and 526 DAT −ve patients explored the relationship between LA and DAT in SLE. The PP of LA was higher in AIHA +ve than AIHA −ve groups (29.2% vs. 15.7%) (Figure 3C).

### 2.5. Comparative Prevalence of Autoimmune Haemolytic Anaemia in Patients with and without Antiphospholipid Antibodies

**Figure 4:**
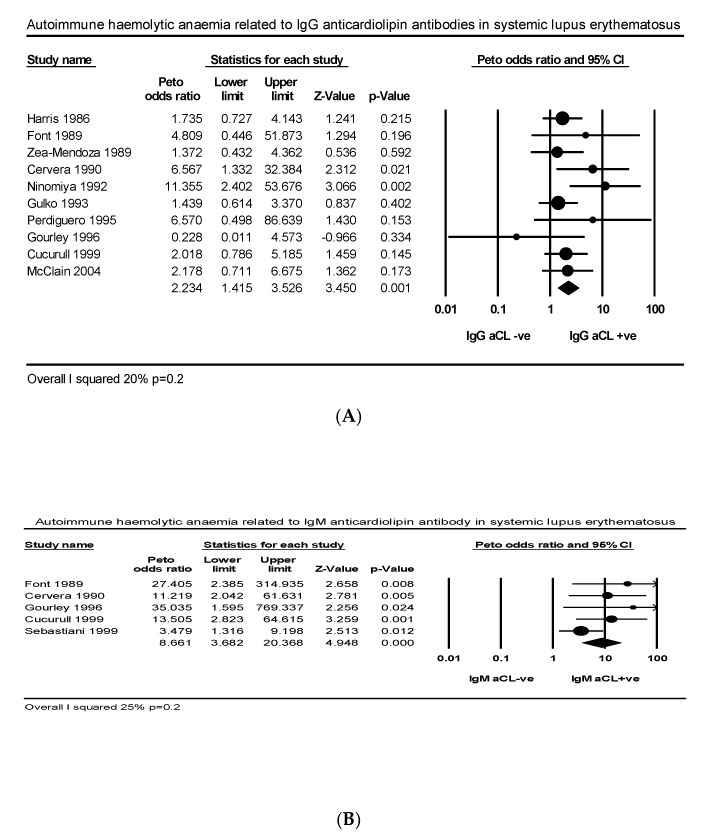
Effect size autoimmune haemolytic anaemia related to (**A**) IgG anticardiolipin, (**B**) IgM anticardiolipin antibody.

Ten articles comprising 352 IgG aCL +ve and 664 IgG aCL −ve SLE patients explored the relationship between AIHA and IgG aCL in SLE. The PP of AIHA was higher in IgG aCL +ve than −ve groups (21.8% vs. 11.1%) with low heterogeneity (*I*^2^ = 20%, *p* = 0.2) (Figure 4A). Five studies comprising 139 IgM aCL +ve and 888 IgM aCL −ve patients explored the relationship between AIHA and IgM aCL in SLE. The PP of AIHA was higher in IgM aCL +ve than −ve groups (18.7% vs. 6.3%) with low heterogeneity (*I*^2^ = 25%, *p* = 0.2) (Figure 4B). Two articles comprising 39 IgM aCL +ve and 69 IgG aCL −ve in PAPS explored the same relationship in PAPS. The PP of AIHA between IgG aCL +ve and −ve groups was similar (12.8% vs. 7.2%) with no heterogeneity. Four studies comprising 75 IgG aβ_2_GPI +ve and 447 IgG aβ_2_GPI −ve patients explored the relationship between AIHA and IgG aβ_2_GPI. The PP of AIHA was higher in IgG aβ_2_GPI +ve than −ve groups (18.6% vs. 7.1%) with wide heterogeneity (*I*^2^ = 58%, *p* = 0.6) (Figure A3). Five articles comprising 271 LA +ve and 950 LA −ve patients explored the relationship between AIHA and LA. The PP of AIHA was greater in LA +ve than LA −ve groups (20% vs. 10%) with mild heterogeneity (*I*^2^ = 29%, *p* = 0.25) (Figure A4).

### 2.6. Comparative Prevalence of Coombs Test in Patients with and without Antiphospholipid Antibodies

Five articles comprising 446 IgG aCL +ve and 417 IgG aCL −ve patients explored the relationship between DAT and IgG aCL. The PP of DAT was higher in IgG aCL +ve than −ve groups (33.6% vs. 11.9%) with wide heterogeneity (*I*^2^ = 72%, *p* = 0.006) (Figure A5). Three articles comprising 51 LA +ve and 117 LA −ve patients explored the relationship between DAT and LA. The PP of AIHA was much higher in LA +ve than −ve groups 27.4% vs. 5.1%, *p* < 0.05) (Figure A6).

### 2.7. Comparative Prevalence of Autoimmune Haemolytic Anaemia and Coombs Test in Relation to Primary and Lupus Related Antiphospholipid Syndrome and to any Thrombosis

**Figure 5:**
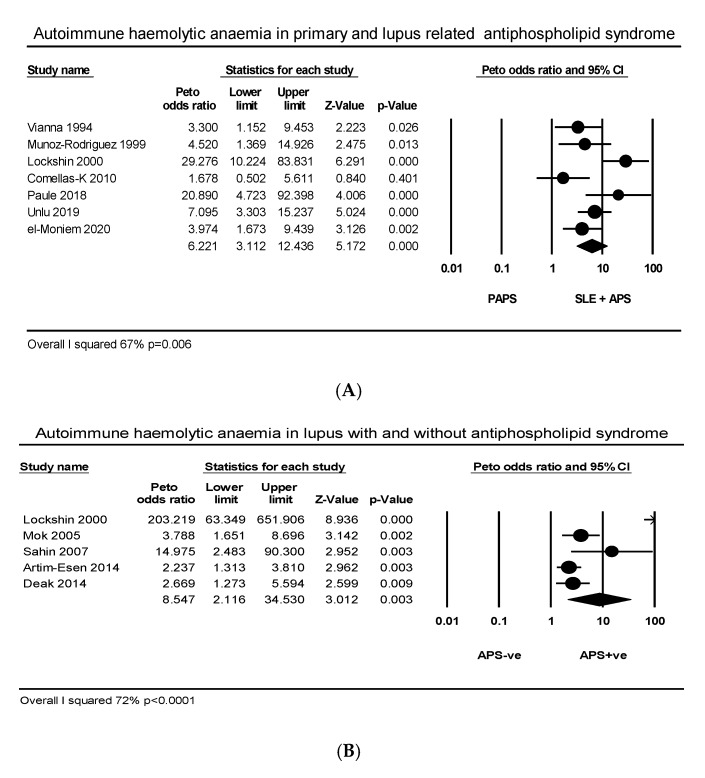

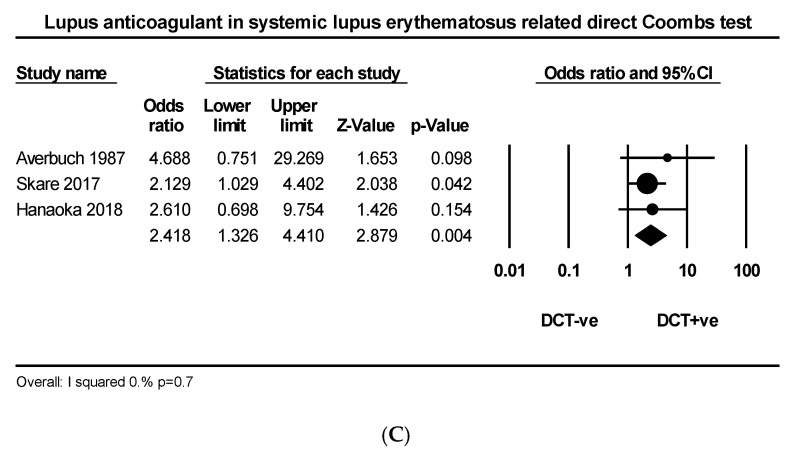
Effect size autoimmune haemolytic anaemia in relation to (**A**) primary and lupus with antiphospholipid syndrome (**B**) lupus with and without antiphospholipid syndrome, (**C**) thrombosis in systemic lupus erythematosus.

Seven articles comprising 482 SLE related and 729 PAPS patients explored the relationship between AIHA and APS. The PP of AIHA was higher in SLE related than in PAPS (22.8% vs. 3.7%) with wide heterogeneity (*I*^2^ = 67%, *p* = 0.006) (Figure 5A). Two articles comprising 41 aPL carriers and 91 primary APS patients explored the relationship between AIHA and APS. The PP of AIHA was higher in aPL carriers than in PAPS (19% vs. 5.4%). Five articles comprising 379 SLE with thrombotic APS and 1117 SLE without thrombosis explored the relationship between AIHA and thrombosis in SLE. The PP of AIHA was greater in APS +ve than APS −ve SLE (23.2% vs. 8.4%) with wide heterogeneity (*I*^2^ = 72%, *p* < 0.0001) due to two studies that over-favored the effect size (Figure 5B). Four articles comprising 123 aPL positive SLE patients with and 607 without any vascular occlusion explored the relationship between AIHA and thrombosis. The PP of AIHA was higher in the thrombotic than the non-thrombotic group (26.8% vs. 10%) with medium heterogeneity (*I*^2^ = 40%, *p* = 0.1 (Figure 5C). Three articles comprising 143 SLE carriers of aPL and 93 PAPS patients explored the relationship between DAT and APS. The PP of DAT was higher in SLE carriers of aPL than in primary APS (37.7% vs. 16%) (*I*^2^ = 70%, *p* = 0.04) (Figure A7).

## 3. Discussion

The studies included in the systematic review were evaluated according to two complementary scenarios. In the first scenario, we determined the PP of aPL positive patients in AIHA, whether SLE related or idiopathic, and in the second scenario we determined the PP of AIHA in patients with aPL, whether affected by SLE and/or APS.

According to the first scenario, the pooled prevalence of patients positive for IgG/IgM aCL and LA were all greater in SLE with AIHA, with a degree of statistical heterogeneity explained by the occasional outlier that favored the effect size in each study. Conversely, the studies on idiopathic AIHA were devoid of heterogeneity. According to the second scenario, AIHA was more common in SLE patients positive for IgG aCL and IgM aCL but less for IgG aβ_2_GPI: AIHA was also more common in LA positive SLE patients with no heterogeneity as in the two complementary studies assessing the relation between DAT and LA. Because of its fluid phase nature, LA may detect a wider repertoire of antibodies than the solid phase immune assay that detects a single antibody, although in both scenarios, IgG/IgM aCL and DAT showed strong reciprocal effect sizes. Altogether, these findings suggest that a clinical manifestation such as AIHA relates prevalently to IgG aCL whereas a laboratory manifestation such as a DAT relates strongly to all aPL.

The varied definitions of AIHA adopted by the authors, the presence of DAT negative patients [65] in few studies may explain some of the observed clinical heterogeneity, though even DAT negative cases may be caused by warm IgM auto-antibodies identifiable by the dual direct antiglobulin test [66]. Alternatively, patients with aPL/APS may yield false positive DATs [67] due to cytophilic or non-specifically adsorbed IgG on the surface of erythrocytes, the eluates of which do not contain any antibody [68]. In SLE, DAT predicts 8-epi-prostaglandin-F2α, a specific marker of lipid peroxidation [69]. In the oxidative environment of SLE and APS [69,70], phospatidylcholine and phosphatidylserine present in the erythrocyte membrane may oxidise contributing to erythrocyte senescence [71,72]: natural IgM autoantibodies may perform housekeeping functions against these phospholipid neo-epitopes [73] but the intra-splenic clearance of senescent erythrocytes is normally mediated by antibody-independent mechanisms, such as the recognition of phosphatidylserine exposed on the outer leaflet of their plasmamembrane by macrophage scavenger receptors [74]. Participation of natural IgM bound to oxidised PS (oxPS) would require complement activation and recognition of iC3b by CR3 and CR4 complement receptors: neither “true” Fc receptors for the IgM isotype, nor mixed Fc receptors for the IgA and IgM isotypes are expressed by human macrophages. On the other hand, natural IgM antibodies bound to oxidized phospholipids may prevent the activation of pro-inflammatory responses of phagocytes via scavenger receptors, such as CD36, CD68, SR-PSOX; these natural antibodies may cross react with aPL as eluates from the erythrocyte of SLE patients with aPL revealed strong cardiolipin [75] and oxidated phospatidylcholine reactivity [76].

Also, the relation between AIHA and thrombosis also offers several scenarios: AIHA was much more common in SLE related APS than in SLE without APS or PAPS. However, these studies comprised not only patients with thrombosis but also patients with obstetric morbidity, the presence of which may have weakened the effect size between AIHA and thrombosis. Nevertheless, there was a greater PP of AIHA in thrombotic rather than non-thrombotic SLE patients and to complete this scenario also the DAT was more common in SLE related APS than PAPS.

The relationship between aPL and AIHA may rely on several different mechanisms related to erythrocyte antigenicity. These may be either proteins/glycoproteins or carbohydrate moieties positioned on glycoproteins and glycolipids [77]: proteins/glycoprotein moieties induce IgG1 and IgG3 subclass response which is T-cell-dependent, whereas carbohydrate moieties induce mostly an IgG2 subclass response which is T-cell-independent [78,79,80].

Shortened erythrocyte survival also depends upon the IgG subclass: IgG1 followed by IgG3 are more efficient than IgG2 and IgG4: the former bind avidly to C1q and are strong complement activators, IgG2 is a poor complement activator, and IgG4 does not activate complement because it does not bind to C1q [81]. In APS, the IgG2 and the IgG3 subclasses [82] are strongly associated with thrombosis [83,84].

Once reached a critical amount on the erythrocyte membrane aPL may activate complement leading to erythrocyte destruction; with regards to hemolysis the effect size was always greater for IgM aCL than for IgG aCL, in keeping with the fact that the IgM isotype is a powerful complement activator, to an even higher extent than the most abundant IgG1 and IgG2 isotypes, but it is not usually found on the erythrocyte surface by DAT in warm AIHA. Low serum complement occurs in 38% of SLE but only in 9% of primary APS patients and it is associated with AIHA in SLE related but not in PAPS [84]. Compared to normal and thrombotic controls, patients with SLE related APS display a higher amount of erythrocyte bound C4d [85]. Moreover, the annexin V binding test revealed that almost 50% of SLE erythrocytes express phosphatidylserine on their surface that is known to support thrombin generation in vitro [75].

Coagulation and complement functions may proceed simultaneously in APS [86]. As part of this cross talk, C5a engages its counter receptor on neutrophils and induces tissue factor release [87] that in turn catalyzes factor X to its active form promoting thrombin generation, whereas downstream of C5a, the C5b–9 membrane attack complex induce platelet and endothelial cell activation [81].

However, we must remember that intravascular hemolysis mediated by the terminal complement pathway is not as prominent as reticulo-endothelial cell phagocytosis of C3b opsonized erythrocytes, that is the main mechanism for warm autoimmune haemolysis.

Summing up the above scenarios it is likely that classical and alternative complement pathways as well as coagulation activation occur in SLE with APS and thrombosis, while complement activation on its own occurs in SLE with or without aPL and coagulation activation only in APS. This would explain the decreasing frequency of AIHA through the three conditions mentioned, though there is recent evidence that complement activation may also occur in APS [88].

Limitations of our meta-analysis include: (1) variability in study sizes, (2) few studies on PAPS, (3) expression of data as frequency of aPL positive participants rather than average antibody titres in most articles, and hence (4) no relation between aPL titers with severity of haemolysis or intensity of DAT positivity, as well as (5) a lack of follow up in patients with isolated DAT positivity to verify whether they do develop AIHA.

Nevertheless, our meta-analysis clearly demonstrates an intimate relationship between aPL and AHIA: the pooled prevalence of patients with AIHA ranges between 23% and 26% in SLE with APS, not much different from the 22.4% deriving from a meta-analysis published in abstract form in 2015 [89]. Likewise, our 8.4% PP from our aPL +ve SLE patients equals the PP of their aPL −ve patients, probably because the authors included amongst their SLE related APS also aPL +ve patients. Once split into individual aPL, their OR for LA was 4.58 (95% CI 2.62–8.04), ours was twice as much when comparing the PP of LA in SLE with and without AIHA (OR 8.3, 95% CI 1.52–45.39) whereas the comparative figures for AIHA in LA +ve and LA −ve SLE patients was twice as low (OR 2.10 95% CI 1.38–3.18). Their risk of AIHA in IgG aCL (OR 2.27, 95% CI 1.71–3.00) was slightly higher than ours (OR 2.23, 95% CI 1.41–3.52) whereas their risk of AIHA in IgM (OR 2.89, 95% CI 2.16–3.87) was almost four times lower than ours (OR 8.66, 95% CI 3.68–20.36). These differences are likely due to the different pooling of data and outcomes in our meta-analysis.

Moreover, the 23–26% AIHA in SLE with APS, the 8.4% in SLE with aPL, and the 4% in PAPS, are in keeping with the inclusion of AIHA in the classification criteria for SLE but not for APS. Likewise, the pooled prevalence of DAT associated with aPL in SLE was around 37% but only 16% in PAPS. Interestingly, AIHA predicts mortality in the first year after diagnosis of SLE and during follow up [90] whereas an isolated DAT predicts disease activity [47,55]. Because disease activity relates to oxidative stress [69], the question arises as to whether an isolated DAT, particularly when caused by an IgM, represents a natural or a pathogenic antibody. In fact, the majority of circulating IgM are polyspecific natural auto-antibodies, secreted in a T-cell-independent manner and encoded by multiple germ-line variable region genes, with little somatic mutation and few non-templated insertions; the minority of circulating IgM are immune IgM produced by B cells selected for antigen-specificity, usually following exposure to pathogens [91]. Thus, natural and (auto)immune IgM may coexist in the same individual. To conclude, the meta-analysis finds a consistent relationship between aPL and AHIA (IgM aCL > IgG aCL > LA > IgG aβ_2_GPI) as well as between AIHA and thrombosis, particularly in patients with SLE: while plausible thrombogenic pathways linking AIHA and SLE do exist, the intimate nature of the reaction between different aPL isotypes and erythrocyte structures remains unclear and warrants further research

## 4. Materials and Methods

### 4.1. Search Strategy and Selection Criteria

Two authors carried out the systematic review according to the PRISMA guidelines [92] was carried out by two searching the electronic databases MEDLINE and EMBASE from inception to February 2020 for the following terms: “haemolysis” OR “haemolytic anaemia” OR “direct antiglobulin test” OR “direct Coombs test” and “anticardiolipin” OR “anti-beta 2-glycoprotein-I” OR “antiphospholipid syndrome”, OR “lupus anticoagulant” OR “lupus inhibitor”. The search yielded 3106 records plus another 12 found amongst the references screened for inclusion in the systematic review. The records were fed into EndNote that removed the duplicates leaving 1177 records. The same authors plus a third screened the records for relevancy and excluded 1049 (including 54 dealing with microangiopathic haemolytic anaemia and 59 case reports), thus leaving 128 eligible articles (Figure 1)

### 4.2. Criteria for Selecting Articles

Inclusion criteria were: (1) case-control and/or cohort observational studies investigating relevant adult populations: (a) the different titers or frequency aPL between patient and control groups with AIHA or with a positive DAT, (b) the different titers or frequency of aPL between AIHA and/or DAT patients positive and negative for AIHA, and (c) the different frequency of AIHA and/or DAT in aPL positive or negative patients; (2) aPL tested by immune or clotting assays; (3) articles written in English, French, and Spanish. We chose the highest-quality study if two or more studies investigated the same population. Exclusion criteria were: (1) prevalence studies only, (2) paediatric populations, (3) catastrophic antiphospholipid syndrome, (4) studies not reporting the relationship between aPL and AIHA, (5) non-original research, (6) articles not written in the languages indicated in the inclusion criteria

### 4.3. Evaluation of the Quality of the Studies

The Newcastle Ottawa Quality Assessment Scale for case-control and cohort studies was employed to assess the quality of the relevant studies [93]. The final score may range between 0 and 8, and results from the sum of the ratings of three major domains: case/controls selection, their comparability, and ascertainment of their exposure to the outcome of interest.

### 4.4. Outcome Measures

The primary outcomes were: (1) the comparative pooled prevalence (PP) of different aPL in participants with and without AIHA or DAT, (2) the comparative pooled prevalence of AIHA or DAT in participants with and without aPL and the standardized mean difference of aPL titres between the groups indicated in number one and two. The secondary outcomes, where possible, were: (1) the comparative pooled prevalence of vascular occlusions in participants positive or negative for aPL plus AIHA or aPL plus DAT, (2) the pooled prevalence of aPL plus AIHA or aPL plus DAT in participants with or without vascular occlusions, and (3) the standardized mean difference of the titers of aPL between participants with and without AIHA or DAT.

### 4.5. Statistical Analysis

**Figure A1:**
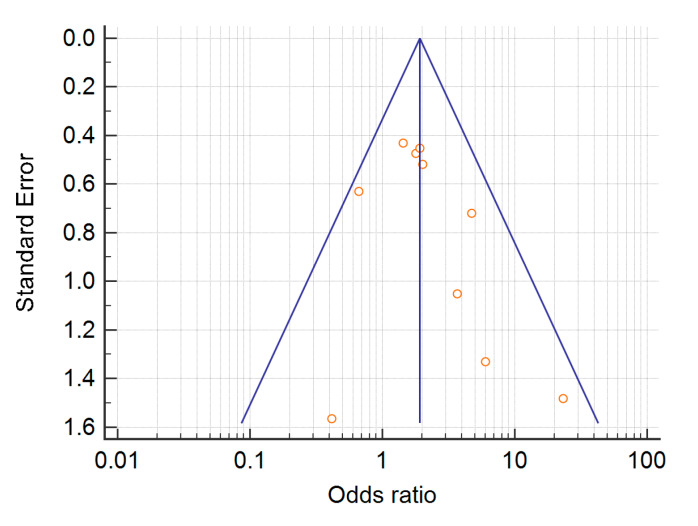
Funnel plot on 10 articles having the same outcome.

**Figure A2:**
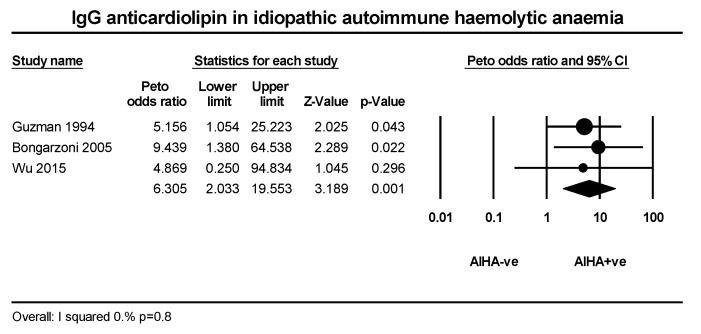
Effect size of IgG anticardiolipin in idiopathic autoimmune haemolytic anaemia.

**Figure A3:**
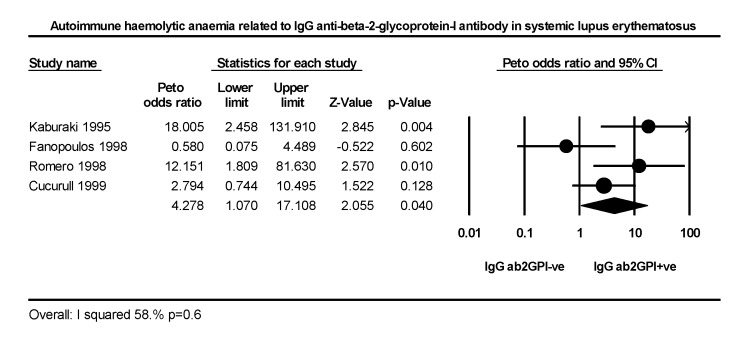
Effect size autoimmune haemolytic anaemia related to IgG anti beta2 glycoprotein-I in systemic lupus erythematosus.

**Figure A4:**
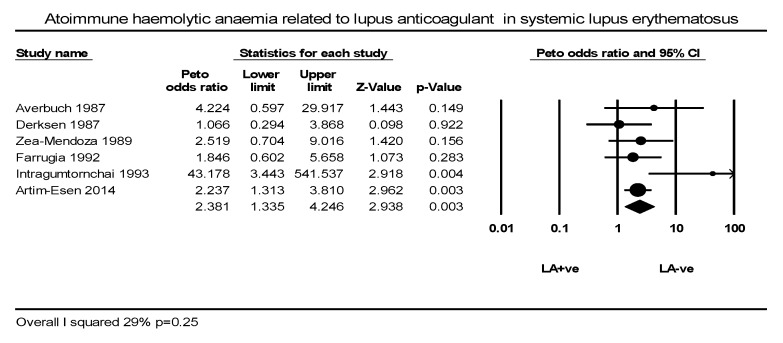
Effect size autoimmune haemolytic anaemia related to lupus anticoagulant in systemic lupus erythematosus.

**Figure A5:**
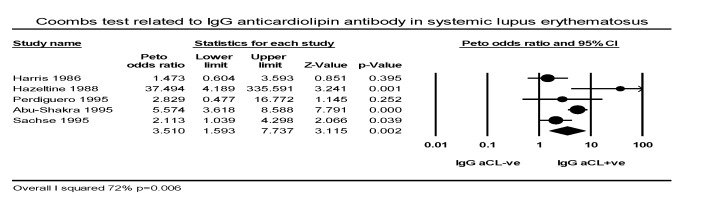
Effect size of antiglobulin test related to IgG anticardiolipin Antibody in systemic lupus erythematosus.

**Figure A6:**
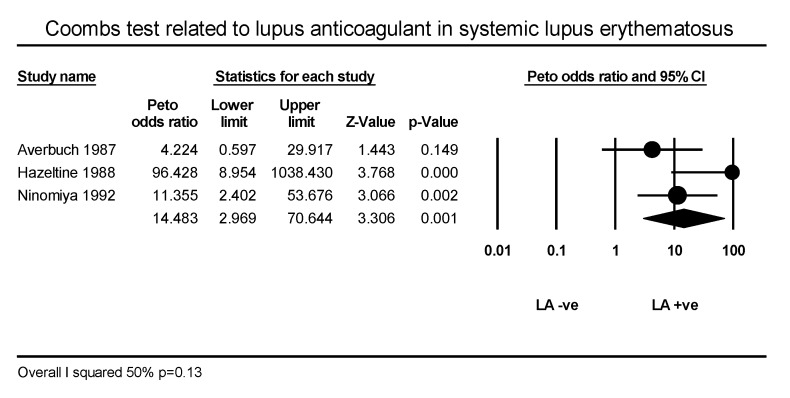
Effect size of antiglobulin test related to lupus anticoagulant in systemic lupus erythematosus.

**Figure A7:**
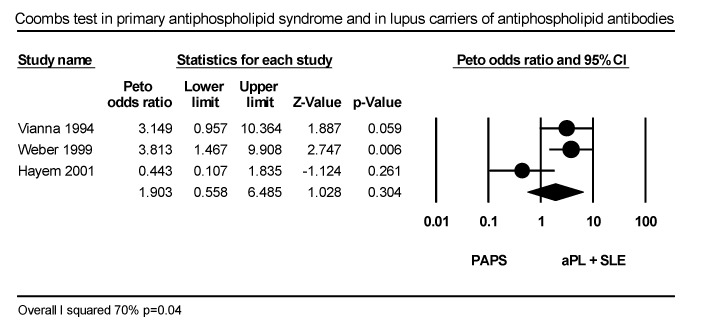
Effect size of antiglobulin test between primary antiphospholipid syndrome and lupus carriers of antiphospholipid antibodies.

As all the included studies included had an observational design with no planned exposure, we employed: (1) random effects meta-analyses for categorical outcomes, (2) Peto’s odds ratio for rare events to compare the pooled frequencies between groups [94], (3) I^2^ statistics to evaluate statistical heterogeneity. Heterogeneity was nil when I^2^ equaled 0%, low when I^2^ was less than 25%, moderate with I^2^ between 25% and 50% and high with I^2^ greater than 50%. Ten studies having similar outcomes yielded a funnel plot showing a slight publication bias due to imprecise study effect [95] (Figure A1) but we did not rely on this result, as funnel plots may be misleading when performed on a small number of studies [96,97]. Comprehensive Meta-Analysis (Biostat, Englewood, New Jersey, USA) was used for the statistical analysis, and the inter-rater agreement of two investigators was assessed by Cohen’s kappa.

## Figures and Tables

**Table 1:** Demographics of studies included in the systematic review and meta-analysis.

**(A)**			**Participants**																
**Studies on SLE/APS**			**SLE**			**PAPS/APL**	**Other**							**aCL**		**aβ_2_GPI**			
**Reference**	**Country**	**Study Type**	**SLE**	**aPL**	**APS**			**Total**	**M/F**	**Assessed**	**Age**	**AIHA**	**DAT**	**IgG**	**IgM**	**IgG**	**IgM**	**LA**	**NOS**
			**No**	**No**	**No**	**No**	No	No	No	No	Mean ± SD/median (range)	No	No	No	No	No	No	No	
[6]	USA	CHT	180				120	300	19/102	121	34 (17–74)		27	74					7
[7]	Israel	CHT	36					36	4/32	36	40 ± 19	8	8					7	6
[8]	Holland	CHT	74				51	112		74		15						19	6
[9]	Mexico	CHT	12					12	0/12	12	26 ± 9		9	7	7			3	7
[10]	Canada	CHT	51	14				65	4/61	65	43	4	4	13				10	8
[11]	Mexico	CHT	500	y	y			500	50/450	500	34 (12–70)	25		200	166				7
[12]	Spain	CC	46	9	5		107	167	42/125	60		4		14	13				6
[13]	Spain	CHT	88	y	y			88		88		14		28				24	5
[14]	Israel	CHT	15				33	48		40		1	12					40	5
[15]	Spain	CHT	64	36				100	7/93	100		9		24	20			30	7
[16]	Australia	CHT	111	y	y			111	6/105	111	15–70		42	31	21				6
[17]	Spain	CHT				23		23	4/19	23	29 ± 8	3		11	7			18	5
[18]	USA	CHT	32	20	13			65	41/24	65	29 ± 12	16						33	6
[19]	Singapore	CHT	46	y				46	46 F	46		17		5	11				5
[20]	Japan	CHR	77	272				349		349			14	41	4			26	4
[21]	USA	CHT	139	y	y			139	32/107	139	33 ± 16	27		57					7
[22]	Thailand	CHT	75	16				91		91		4						14	5
[23]	MC	CHT			56	58		114	22/92	114	34 ± 14	16	12						7
[24]	Germany	CHT	124	244				368	36/332	368	48 (12–76)		27	168					8
[25]	Canada	CHT	206	184				390	47/343	390	32 (7–83)		118	184					6
[26]	Japan	CHT	119	21				140	16/124	140	34 ± 12	8		61		21			6
[27]	Spain	CHT	16	7				23	0/23	23	28	5	9	7	7				6
[28]	Ireland	CHT	53	42				95	10/85	95	13–70	4	1	30	27				6
[29]	Germany	CC	96	y			152	248		96		22	22	53 ± 57	28 ± 32				7
[30]	USA	CHT	38	y	10			48	10/38	28		6		16	10	11	9		6
[31]	UK	CHT	78		40			118	4/114	118	40 (10–71)	8				20			5
[32]	MC	CHT	258	80	y			258	34/224	258	29 (4–70)	25		60	20	27	23		7
[33]	Europe	MCT	574	y				574	44/503	574	38 ± 13	48		119	73	110	109		7
[34]	Spain	CHT			38	62		100	14/86	100	36 (13–79)	13		74	32				7
[35]	Switzerland	CHT			86	22		108	14/94	108	42 ± 13		44	22				48	8
[36]	Greece	CHT	132	y				132	10/122	55		19		13		12			7
[37]	USA	CHT	168		26	49		243	14/229	206	41 ± 12	26		130	50			136	6
[38]	USA	CHT			34	48		82	17/65	77	27 (21–32)	12				50			7
[39]	France	CHT	80			13	18	111	10/101	111	29 ± 12		29	54		21		35	7
[40]	Spain	CHT	600	y	y			600	67/533	600	31 ± 6	50		82	51				7
[41]	USA	CHT	106	24				130	46/84	130	29.3	25		24					7
[42]	MC	CHT	385	157	83			385	23/249	385	38 ± 10	32		73					7
[43]	MCT	CHT				308		308	48/260	308	40 ± 11	32		192		217		239	6
[44]	Turkey	CHT	44		15			59	6/53	59	35 ± 11	7		8	13	21	11	10	7
[45]	Thailand	CHT		41	26			67	2/65	67	30 ± 10	28		39		59		18	8
[46]	USA	CHT	909	y				909	0/909	909		76		272	79				7
[47]	USA	CHT	628	y	y			628	62/566	628	34 ± 12	65		149				32	7
[48]	Brazil	CHT	362	y	y			362	21/341	362	37 ± 12	44		39	39			20	8
[49]	Mexico	CHT				25/30		55	11/44	55	40 ± 13	14		37	50	30	37	23	7
[50]	Hungary	CHT	119	53	52			224	20/204	224	49 (20–92)	45		215					6
[51]	Turkey	CHT	852	y	y			852	110/742	852	31 ± 12	93	175	175				175	7
[52]	Brasil	CHT	460	y	y			460	30/430	460	16–88	39		55	53			59	7
[53]	MC	CHT	833	604				1437	58/1290	1437	30 ± 12	79		340	340				8
[54]	Brasil	CHT	373	y				373	26/347	373	42 (22–51)	48		64	49			56	7
[55]	Japan	CHT	182	y				182	22/160	182	45 ± 14	10						39	7
[56]	France	CHT			72	28		100	18/82	100	34 (28–39)	9	29	87	87	48	48	90	8
[57]	Turkey	CHT	725	y	125			850		850		93	174	24	169			91	6
[58]	MC	CHT		y	y	y/y		623	164/459	623	44 ± 12	64		714	446	530	228	834	8
[59]	Mexico	CHT				95			28/67	95	44 ± 14	8		54	30	41	20	58	7
[60]	Egypt	CHT	42		106				13/135	148	23 ± 7	32		148					6
**(B)**			**Participants**																
**Studies on AIHA**	**Country**	**Study Type**	**AIHA**			**Non AIHA**	**CTR**							**aCL**		**aβ_2_GPI**			
**Reference**			**ID**	**SLE**	**Other**			**Total**	**M/F**	**Assessed**	**Age**	**AIHA**	**DAT**	**IgG**	**IgM**	**IgG**	**IgM**	**LA**	**NOS**
			No	No	No	No	No												
[61]	Mexico	CC	18			14		32	19/16	32	33 ± 16	18	14	7	4				7
[62]	USA	CHT	26	3	1			30	11/19	30	43 (21–76)	30		5	12			9	7
[63]	Italy	CC	21		26		42	89	9/12	63		21		5				6	8
[64]	China	CC	19	18				37	15/22	37	48 ± 18	37	37	11					6

Abbreviations. M/F: male/female; AIHA: autoimmune haemolytic anaemia; DAT: direct antiglobulin test; aCL: anticardiolipin; aβ_2_GPI: anti-beta-2-glycoprotein-I; LA: lupus anticoagulant; NOS: Newcastle-Ottawa Scoring; No; number; SLE: systemic lupus erythematosus; CHT: cohort; APS: antiphospholipid syndrome; PAPS: primary antiphospholipid syndrome; CC: case control.

## Abbreviations

APL	antiphospholipid antibodies
APS	antiphospholipid syndrome
SLE	systemic lupus erythematosus
AIHA	autoimmune haemolytic anaemia
DAT	direct antiglobulin test
aCL	anticardiolipin
PP	pooled prevalence
LA	lupus anticoagulant
aβ_2_GPI	anti-beta-2-glycoprotein-I

## References

[1] Limper M., Scirè C.A., Talarico R., Amoura Z., Avcin T., Basile M., Burmester G., Carli L., Cervera R., Costedoat-Chalumeau N. (2018). Antiphospholipid syndrome: State of the art on clinical practice guidelines. RMD Open.

[2] Hill Q.A., Stamps R., Massey E., Grainger J.D., Provan D., Hill A. (2017). British Society for Haematology. The diagnosis and management of primary autoimmune haemolytic anaemia. Br. J. Haematol..

[3] Aringer M., Costenbader K., Daikh D., Brinks R., Mosca M., Ramsey-Goldman R., Smolen J.S., Wofsy D., Boumpas D.T., Kamen D.L. (2019). European League Against Rheumatism/American College of Rheumatology classification criteria for systemic lupus erythematosus. Ann. Rheum. Dis..

[4] Petri M., Orbai A.M., Alarcón G.S., Gordon C., Merrill J.T., Fortin P.R., Bruce I.N., Isenberg D., Wallace D.J., Nived O. (2012). Derivation and validation of the Systemic Lupus International Collaborating Clinics classification criteria for systemic lupus erythematosus. Arthritis Rheum..

[5] Pignatelli P., Ettorre E., Menichelli D., Pani A., Violi F., Pastori D. (2020). Seronegative antiphospholipid syndrome: Refining the value of “non-criteria” antibodies for diagnosis and clinical management. Haematologica.

[6] Harris E.N., Chan J.K., Asherson R.A., Aber V.R., Gharavi A.E., Hughes G.R. (1986). Thrombosis, recurrent fetal loss, and thrombocytopenia. Predictive value of the anticardiolipin antibody test. Arch. Intern. Med..

[7] Averbuch M., Koifman B., Levo Y. (1987). Lupus anticoagulant, thrombosis and thrombocytopenia in systemic lupus erythematosus. Am. J. Med. Sci..

[8] Derksen R.H., Bouma B.N., Kater L. (1987). The prevalence and clinical associations of the lupus anticoagulant in systemic lupus erythematosus. Scand. J. Rheumatol..

[9] Delezé M., Oria C.V., Alarcón-Segovia D. (1988). Occurrence of both hemolytic anemia and thrombocytopenic purpura (Evans’ syndrome) in systemic lupus erythematosus. Relationship to antiphospholipid antibodies. J. Rheumatol..

[10] Hazeltine M., Rauch J., Danoff D., Esdaile J.M., Tannenbaum H. (1988). Antiphospholipid antibodies in systemic lupus erythematosus: Evidence of an association with positive Coombs’ and hypocomplementemia. J. Rheumatol..

[11] Alarcón-Segovia D., Delezé M., Oria C.V., Sánchez-Guerrero J., Gómez-Pacheco L., Cabiedes J., Fernández L., Ponce de León S. (1989). Antiphospholipid antibodies and the antiphospholipid syndrome in systemic lupus erythematosus. A prospective analysis of 500 consecutive patients. Medicine (Baltimore).

[12] Font J., Cervera R., Lopez-Soto A., Pallares L., Bosch X., Ampurdanes S., Casals F.J., Ingelmo M. (1989). Anticardiolipin antibodies in patients with autoimmune diseases: Isotype distribution and clinical associations. Clin. Rheumatol..

[13] Zea Mendoza A., Rodríguez García A., Irigoyen Oyarzábal M.V., Vázquez Díaz M., Pardo Vigo A., Mampaso F.M., Ortuño Mirete J. (1989). Antiphospholipid antibodies in systemic lupus erythematosus: Incidence, significance and relation to lupus nephritis. Med. Clin. (Barc.).

[14] Kornberg A., Silber L., Yona R., Kaufman S. (1989). Clinical manifestations and laboratory findings in patients with lupus anticoagulants. Eur. J. Haematol..

[15] Cervera R., Font J., López-Soto A., Casals F., Pallarés L., Bové A., Ingelmo M., Urbano-Márquez A. (1990). Isotype distribution of anticardiolipin antibodies in systemic lupus erythematosus: Prospective analysis of a series of 100 patients. Ann. Rheum. Dis..

[16] Wang Y., Schrieber L., Cohen M.G., Furphy L., Webb J., Chivers T., Pollard K.M. (1990). Antiphospholipid antibodies in systemic lupus erythematosus: Clinical and laboratory associations in 111 patients. Rheumatol. Int..

[17] Font J., López-Soto A., Cervera R., Balasch J., Pallarés L., Navarro M., Bosch X., Ingelmo M. (1991). The ‘primary’ antiphospholipid syndrome: Antiphospholipid antibody pattern and clinical features of a series of 23 patients. Autoimmunity.

[18] Farrugia E., Torres V.E., Gastineau D., Michet C.J., Holley K.E. (1992). Lupus anticoagulant in systemic lupus erythematosus: A clinical and renal pathological study. Am. J. Kidney Dis..

[19] Fong K.Y., Loizou S., Boey M.L., Walport M.J. (1992). Anticardiolipin antibodies, haemolytic anaemia and thrombocytopenia in systemic lupus erythematosus. Br. J. Rheumatol..

[20] Ninomiya C., Taniguchi O., Kato T., Hirano T., Hashimoto H., Hirose S. (1992). Distribution and clinical significance of lupus anticoagulant and anticardiolipin antibody in 349 patients with systemic lupus erythematosus. Intern. Med..

[21] Gulko P.S., Reveille J.D., Koopman W.J., Burgard S.L., Bartolucci A.A., Alarcón G.S. (1993). Anticardiolipin antibodies in systemic lupus erythematosus: Clinical correlates, H.LA associations, and impact on survival. J. Rheumatol..

[22] Intragumtornchai T., Akkawat B., Mahasandana S., Watananukul P., Deesomchok U. (1993). Lupus anticoagulant in Thai systemic lupus erythematosus patients. Southeast Asian J. Trop. Med. Public Health.

[23] Vianna J.L., Khamashta M.A., Ordi-Ros J., Font J., Cervera R., Lopez-Soto A., Tolosa C., Franz J., Selva A., Ingelmo M. (1994). Comparison of the primary and secondary antiphospholipid syndrome: A European Multicenter Study of 114 patients. Am. J. Med..

[24] Sachse C., Lüthke K., Hartung K., Fricke M., Liedvogel B., Kalden J.R., Peter H.H., Lakomek H.J., Henkel E., Deicher H. (1995). Significance of antibodies to cardiolipin in unselected patients with systemic lupus erythematosus: Clinical and laboratory associations. The S.LE Study Group. Rheumatol. Int..

[25] Abu-Shakra M., Gladman D.D., Urowitz M.B., Farewell V. (1995). Anticardiolipin antibodies in systemic lupus erythematosus: Clinical and laboratory correlations. Am. J. Med..

[26] Kaburaki J., Kuwana M., Yamamoto M., Kawai S., Matsuura E., Ikeda Y. (1995). Clinical significance of phospholipid-dependent anti-beta 2-glycoprotein I (beta 2-G.PI) antibodies in systemic lupus erythematosus. Lupus.

[27] Perdiguero M., Boronat M., Marco P., Rivera F. (1995). The role of antiphospholipid antibodies in lupus nephropathy. Nephron.

[28] Gourley I.S., McMillan S.A., Bell A.L. (1996). Clinical features associated with a positive anticardiolipin antibody in Irish patients with systemic lupus erythematosus. Clin. Rheumatol..

[29] Lang B., Straub R.H., Weber S., Röther E., Fleck M., Peter H.H. (1997). Elevated anticardiolipin antibodies in autoimmune haemolytic anaemia irrespective of underlying systemic lupus erythematosus. Lupus.

[30] Fanopoulos D., Teodorescu M.R., Varga J., Teodorescu M. (1998). High frequency of abnormal levels of IgA anti-beta2-glycoprotein I antibodies in patients with systemic lupus erythematosus: Relationship with antiphospholipid syndrome. J. Rheumatol..

[31] Romero F.I., Amengual O., Atsumi T., Khamashta M.A., Tinahones F.J., Hughes G.R. (1998). Arterial disease in lupus and secondary antiphospholipid syndrome: Association with anti-beta2-glycoprotein I antibodies but not with antibodies against oxidized low-density lipoprotein. Br. J. Rheumatol..

[32] Cucurull E., Espinoza L.R., Mendez E., Molina J.F., Molina J., Ordi-Ros J., Gharavi A.E. (1999). Anticardiolipin and anti-beta2glycoprotein-I antibodies in patients with systemic lupus erythematosus: Comparison between Colombians and Spaniards. Lupus.

[33] Sebastiani G.D., Galeazzi M., Tincani A., Piette J.C., Font J., Allegri F., Mathieu A., Smolen J., de Ramon Garrido E., Fernandez-Nebro A. (1999). Anticardiolipin and anti-beta2G.PI antibodies in a large series of European patients with systemic lupus erythematosus. Prevalence and clinical associations. European Concerted Action on the Immunogenetics of S.LE. Scand. J. Rheumatol..

[34] Muñoz-Rodriguez F.J., Font J., Cervera R., Reverter J.C., Tàssies D., Espinosa G., López-Soto A., Carmona F., Balasch J., Ordinas A. (1999). Clinical study and follow-up of 100 patients with the antiphospholipid syndrome. Semin. Arthritis Rheum..

[35] Weber M., Hayem G., De Bandt M., Seifert B., Palazzo E., Roux S., Kahn M.F., Meyer O. (1999). Classification of an intermediate group of patients with antiphospholipid syndrome and lupus-like disease: Primary or secondary antiphospholipid syndrome?. J. Rheumatol..

[36] Voulgarelis M., Kokori S.I., Ioannidis J.P., Tzioufas A.G., Kyriaki D., Moutsopoulos H.M. (2000). Anaemia in systemic lupus erythematosus: Aetiological profile and the role of erythropoietin. Ann. Rheum. Dis..

[37] Lockshin M.D., Sammaritano L.R., Schwartzman S. (2000). Validation of the Sapporo criteria for antiphospholipid syndrome. Arthritis Rheum..

[38] Tektonidou M.G., Ioannidis J.P., Boki K.A., Vlachoyiannopoulos P.G., Moutsopoulos H.M. (2000). Prognostic factors and clustering of serious clinical outcomes in antiphospholipid syndrome. QJM.

[39] Hayem G., Nicaise-Roland P., Palazzo E., de Bandt M., Tubach F., Weber M., Meyer O. (2001). Anti-oxidized low-density-lipoprotein (OxLDL) antibodies in systemic lupus erythematosus with and without antiphospholipid syndrome. Lupus.

[40] Font J., Cervera R., Ramos-Casals M., García-Carrasco M., Sents J., Herrero C., del Olmo J.A., Darnell A., Ingelmo M. (2004). Clusters of clinical and immunologic features in systemic lupus erythematosus: Analysis of 600 patients from a single center. Semin. Arthritis Rheum..

[41] McClain M.T., Arbuckle M.R., Heinlen L.D., Dennis G.J., Roebuck J., Rubertone M.V., Harley J.B., James J.A. (2004). The prevalence, onset, and clinical significance of antiphospholipid antibodies prior to diagnosis of systemic lupus erythematosus. Arthritis Rheum..

[42] Mok C.C., Tang S.S., To C.H., Petri M. (2005). Incidence and risk factors of thromboembolism in systemic lupus erythematosus: A comparison of three ethnic groups. Arthritis Rheum..

[43] Rottem M., Krause I., Fraser A., Stojanovich L., Rovensky J., Shoenfeld Y. (2006). Autoimmune hemolytic anaemia in the antiphospholipid syndrome. Lupus.

[44] Sahin M., Duzgun N., Tunc S.E., Tutkak H. (2007). Antibodies to beta2-glycoprotein-I: Relation of anticardiolipin antibodies with clinical and laboratory parameters in patients with systemic lupus erythematosus. Clin. Biochem..

[45] Choojitarom K., Verasertniyom O., Totemchokchyakarn K., Nantiruj K., Sumethkul V., Janwityanujit S. (2008). Lupus nephritis and Raynaud’s phenomenon are significant risk factors for vascular thrombosis in SLE patients with positive antiphospholipid antibodies. Clin. Rheumatol..

[46] Jeffries M., Hamadeh F., Aberle T., Glenn S., Kamen D.L., Kelly J.A., Reichlin M., Harley J.B., Sawalha A.H. (2008). Haemolytic anaemia in a multi-ethnic cohort of lupus patients: A clinical and serological perspective. Lupus.

[47] Durán S., Apte M., Alarcón G.S., Marion M.C., Edberg J.C., Kimberly R.P., Zhang J., Langefeld C.D., Vilá L.M., Reveille J.D. (2008). Features associated with, and the impact of, hemolytic anemia in patients with systemic lupus erythematosus: L.X, results from a multiethnic cohort. Arthritis Rheum..

[48] Domiciano D.S., Shinjo S.K. (2010). Autoimmune hemolytic anemia in systemic lupus erythematosus: Association with thrombocytopenia. Clin. Rheumatol..

[49] Comellas-Kirkerup L., Hernández-Molina G., Cabral A.R. (2010). Antiphospholipid—associated thrombocytopenia or autoimmune hemolytic anemia in patients with or without definite primary antiphospholipid syndrome according to the Sapporo revised classification criteria: A 6-year follow-up study. Blood.

[50] Deák M., Bocskai M., Burcsár S., Dányi O., Fekete Z., Kovács L. (2014). Non-thromboembolic risk in systemic lupus erythematosus associated with antiphospholipid syndrome. Lupus.

[51] Artim-Esen B., Çene E., Şahinkaya Y., Ertan S., Pehlivan Ö., Kamali S., Gül A., Öcal L., Aral O., Inanç M. (2014). Cluster analysis of autoantibodies in 852 patients with systemic lupus erythematosus from a single center. J. Rheumatol..

[52] Skare T., Damin R., Hofius R. (2015). Prevalence of the American College of Rheumatology hematological classification criteria and associations with serological and clinical variables in 460 systemic lupus erythematosus patients. Rev. Bras. Hematol. Hemoter..

[53] González-Naranjo L.A., Betancur O.M., Alarcón G.S., Ugarte-Gil M.F., Jaramillo-Arroyave D., Wojdyla D., Pons-Estel G.J., Rondón-Herrera F., Vásquez-Duque G.M., Quintana-López G. (2016). Features associated with hematologic abnormalities and their impact in patients with systemic lupus erythematosus: Data from a multiethnic Latin American cohort. Semin. Arthritis Rheum..

[54] Skare T., Picelli L., Dos Santos T.A.G., Nisihara R. (2017). Direct antiglobulin (Coombs) test in systemic lupus erythematosus patients. Clin. Rheumatol..

[55] Hanaoka H., Iida H., Kiyokawa T., Takakuwa Y., Kawahata K. (2018). A positive direct Coombs’ test in the absence of hemolytic anemia predicts high disease activity and poor renal response in systemic lupus erythematosus. Lupus.

[56] Paule R., Morel N., Le Guern V., Fredi M., Coutte L., Belhocine M., Mouthon L., le Jeunne C., Chauvin A., Piette J.C. (2018). Classification of primary antiphospholipid syndrome as systemic lupus erythematosus: Analysis of a cohort of 214 patients. Autoimmun. Rev..

[57] Artım-Esen B., Çene E., Şahinkaya Y., Erdugan M., Oğuz E., Gül A., Öcal L., İnanç M. (2019). Autoimmune haemolytic anaemia and thrombocytopaenia in a single-centre cohort of patients with systemic lupus erythematosus from Turkey: Clinical associations and effect on disease damage and survival. Lupus.

[58] Unlu O., Erkan D., Barbhaiya M., Andrade D., Nascimento I., Rosa R., Banzato A., Pengo V., Ugarte A., Gerosa M. (2019). AntiPhospholipid Syndrome Alliance for Clinical Trials and InternatiOnal Networking Investigators. The Impact of Systemic Lupus Erythematosus on the Clinical Phenotype of Antiphospholipid Antibody-Positive Patients: Results from the AntiPhospholipid Syndrome Alliance for Clinical Trials and InternatiOnal Clinical Database and Repository. Arthritis Care Res. (Hoboken).

[59] Núñez-Álvarez C.A., Hernández-Molina G., Bermúdez-Bermejo P., Zamora-Legoff V., Hernández-Ramírez D.F., Olivares-Martínez E., Cabral A.R. (2019). Prevalence and associations of anti-phosphatidylserine/prothrombin antibodies with clinical phenotypes in patients with primary antiphospholipid syndrome: aP.S/P.T antibodies in primary antiphospholipid syndrome. Thromb. Res..

[60] El-Moniem G.A., El-Garf K., Sobhy N., Mohamed S. (2020). Characterization of the clinical and laboratory features of primary and secondary antiphospholipid syndrome in a cohort of Egyptian patients. Curr. Rheumatol. Rev..

[61] Guzmán J., Cabral A.R., Cabiedes J., Pita-Ramirez L., Alarcón-Segovia D. (1994). Antiphospholipid antibodies in patients with idiopathic autoimmune haemolytic anemia. Autoimmunity.

[62] Pullarkat V., Ngo M., Iqbal S., Espina B., Liebman H.A. (2002). Detection of lupus anticoagulant identifies patients with autoimmune haemolytic anaemia at increased risk for venous thromboembolism. Br. J. Haematol..

[63] Bongarzoni V., Annino L., Roveda A., Amendolea M.A., Tirindelli M.C., Avvisati G. (2005). Risk of thromboembolism in patients with idiopathic autoimmune hemolytic disease and antiphospholipid antibodies: Results from a prospective, case-control study. Haematologica.

[64] Wu B., Wang W., Zhan Y., Li F., Zou S., Sun L., Cheng Y. (2015). CXCL13, CCL4, and sTNFR as circulating inflammatory cytokine markers in primary and SLE-related autoimmune hemolytic anemia. J. Transl. Med..

[65] Barcellini W., Fattizzo B., Zaninoni A., Radice T., Nichele I., Di Bona E., Lunghi M., Tassinari C., Alfinito F., Ferrari A. (2014). Clinical heterogeneity and predictors of outcome in primary autoimmune hemolytic anemia: A G.IM.EM.A study of 308 patients. Blood.

[66] Bartolmas T., Salama A. (2010). A dual antiglobulin test for the detection of weak or non-agglutinating immunoglobulin M warm autoantibodies. Transfusion.

[67] Win N., Islam S.I., Peterkin M.A., Walker I.D. (1997). Positive direct antiglobulin test due to antiphospholipid antibodies in normal healthy blood donors. Vox Sang..

[68] Dubarry M., Charron C., Habibi B., Bretagne Y., Lambin P. (1993). Quantitation of immunoglobulin classes and subclasses of autoantibodies bound to red cells in patients with and without hemolysis. Transfusion.

[69] Ames P.R.J., Alves J., Murat I., Isenberg D.A., Nourooz-Zadeh J. (1999). Oxidative stress in systemic lupus erythematosus and allied conditions with vascular involvement. Rheumatology.

[70] Ames P.R.J., Tommasino C., Alves J., Morrow J.D., Iannaccone L., Fossati G., Caruso S., Caccavo F., Brancaccio V. (2000). Antioxidant susceptibility of pathogenic pathways in subjects with antiphospholipid antibodies: A pilot study. Lupus.

[71] Cabiedes J., Cabral A.R., López-Mendoza A.T., Cordero-Esperón H.A., Huerta M.T., Alarcón-Segovia D. (2002). Characterization of anti-phosphatidylcholine polyreactive natural autoantibodies from normal human subjects. J. Autoimmun..

[72] Greenberg M.E., Sun M., Zhang R., Febbraio M., Silverstein R., Hazen S.L. (2006). Oxidized phosphatidylserine-C.D36 interactions play an essential role in macrophage-dependent phagocytosis of apoptotic cells. J. Exp. Med..

[73] Connor J., Pak C.C., Schroit A.J. (1994). Exposure of phosphatidylserine in the outer leaflet of human red blood cells. Relationship to cell density, cell age, and clearance by mononuclear cells. J. Biol. Chem..

[74] Whelihan M.F., Zachary V., Orfeo T., Mann K.G. (2012). Prothrombin activation in blood coagulation: The erythrocyte contribution to thrombin generation. Blood.

[75] Sthoeger Z., Sthoeger D., Green L., Geltner D. (1993). The role of anticardiolipin autoantibodies in the pathogenesis of autoimmune hemolytic anemia in systemic lupus erythematosus. J. Rheumatol..

[76] Cabral A.R., Cabiedes J., Alarcón-Segovia D. (1990). Hemolytic anemia related to an IgM autoantibody to phosphatidylcholine that binds in vitro to stored and to bromelain-treated human erythrocytes. J. Autoimmun..

[77] Daniels G. (2010). The molecular definition of red cell antigens. ISBT Sci. Ser..

[78] Scott M.G., Shackelford P.G., Briles D.E., Nahm M.H. (1988). Human IgG subclasses and their relation to carbohydrate antigen immunocompetence. Diagn. Clin. Immunol..

[79] Johnson J.L., Jones M.B., Ryan S.O., Cobb B.A. (2013). The regulatory power of glycans and their binding partners in immunity. Trends Immunol..

[80] De Libero G., Mori L. (2014). The T-Cell Response to Lipid Antigens of Mycobacterium tuberculosis. Front. Immunol..

[81] Mathern D.R., Heeger P.S. (2015). Molecules Great and Small: The Complement System. Clin. J. Am. Soc. Nephrol..

[82] Samarkos M., Davies K.A., Gordon C., Walport M.J., Loizou S. (2001). IgG subclass distribution of antibodies against beta (2)-G.P1 and cardiolipin in patients with systemic lupus erythematosus and primary antiphospholipid syndrome, and their clinical associations. Rheumatology.

[83] Arvieux J., Roussel B., Ponard D., Colomb M.G. (1994). IgG2 subclass restriction of anti-beta 2 glycoprotein 1 antibodies in autoimmune patients. Clin. Exp. Immunol..

[84] Ramos-Casals M., Campoamor M.T., Chamorro A., Salvador G., Segura S., Botero J.C., Yagüe J., Cervera R., Ingelmo M., Font J. (2004). Hypocomplementemia in systemic lupus erythematosus and primary antiphospholipid syndrome: Prevalence and clinical significance in 667 patients. Lupus.

[85] Lonati P.A., Scavone M., Gerosa M., Borghi M.O., Pregnolato F., Curreli D., Podda G., Femia E.A., Barcellini W., Cattaneo M. (2019). Blood Cell-Bound C4d as a Marker of Complement Activation in Patients With the Antiphospholipid Syndrome. Front. Immunol..

[86] Ames P.R.J., Alves J.D., Gentile F. (2017). Coagulation and complement in antiphospholipid syndrome. Thromb. Res..

[87] Ritis K., Doumas M., Mastellos D., Micheli A., Giaglis S., Magotti P., Rafail S., Kartalis G., Sideras P., Lambris J.D. (2006). A novel C5a receptor-tissue factor cross-talk in neutrophils links innate immunity to coagulation pathways. J. Immunol..

[88] Zhou Y., Chen P., Li Y. (2019). Association between antiphospholipid antibodies and factor Bb in lupus nephritis patients with glomerular microthrombosis. Int. J. Rheum. Dis..

[89] Unlu O., Wahl D., Zuily S. (2015). Increased Risk of Hemolytic Anemia Associated with Antiphospholipid Antibodies in Patients with Systemic Lupus Erythematosus: A Systematic Review and Meta-Analysis [abstract]. Arthritis Rheumatol..

[90] Kasitanon N., Magder L.S., Petri M. (2006). Predictors of survival in systemic lupus erythematosus. Medicine (Baltimore).

[91] Wang H., Coligan J.E., Morse H.C. (2016). Emerging Functions of Natural IgM and Its Fc Receptor FCMR in Immune *Homeostasis*. Front. Immunol..

[92] Liberati A., Altman D.G., Tetzlaff J., Mulrow C., Gøtzsche P.C., Ioannidis J.P.A., Clarke M., Devereaux P.J., Kleijnen J., Moher D. (2009). The PRISMA statement for reporting systematic reviews and meta-analyses of studies that evaluate health care interventions: Explanation and elaboration. Ann. Intern. Med..

[93] Wells G.A., Shea B., O’Connell D., Peterson J., Welch V., Losos M., Tugwell P. The Newcastle-Ottawa Scale (NOS) for Assessing the Quality of Nonrandomized Studies in Meta-Analyses. http://www.ohri.ca/programs/clinical_epidemiology/oxford.htm.

[94] Catharina Brockhaus A., Grouven U., Bender R. (2016). Performance of the Peto’s odds ratio compared to the usual odds ratio estimator in the case of rare events. Biom. J..

[95] Choi S.W., Lam D.M.H. (2016). Funnels for publication bias—have we lost the plot?. Anaesthesia.

[96] Tang J.L., Liu J.L. (2000). Misleading funnel plot for detection of bias in meta-analysis. Clin. Epidemiol..

[97] Lau J., Ioannidis J.P.A., Terrin N., Schmid C.H., Olkin I. (2006). The case of the misleading funnel plot. Br. Med. J..

